# The prevalence of mental disorders among young people in europe

**DOI:** 10.1192/j.eurpsy.2021.596

**Published:** 2021-08-13

**Authors:** R. Sacco, N. Camilleri, K. Umla-Runge

**Affiliations:** 1 Child And Young People’s Services, Malta Mental Health Services, Pieta, Malta; 2 Psychiatry, Cardiff University, Cardiff, United Kingdom

**Keywords:** Child, adolescent, prevalence, Europe

## Abstract

**Introduction:**

This systematic review and meta-analysis fills a lacuna in the existing literature on the prevalence of mental disorders (MD) among young people (YP) in Europe.

**Objectives:**

This study sets out to estimate the pooled prevalence (PP) of ASD, ADHD, Conduct Disorder (CD), Oppositional Defiant Disorder (ODD), Anxiety Disorder (AD), Depressive Disorder (DD), Eating Disorder (ED), Substance Use Disorders (SUD) and the PP of any of these MD, among 5-to-18-year-old YP living in Europe, based on prevalence rates established in the last five years (LFY).

**Methods:**

A search strategy was created following the SPIDER model and conducted on three databases. Studies were also identified from reference lists and grey literature. Eligible studies were evaluated for bias. Trends of prevalence rates across countries, gender and level of education were analysed. The random effects pooled prevalence rate (REPPR) for each MD and for any MD was calculated.

**Results:**

The European REPPR for any mental disorder among YP is 15.5%, translating to almost 1 in 5 YP. ADs are the most common group of MDs with a REPPR of 7.9%, followed by ADHD, ODD, MDD and CD, with REPPR of 2.9%, 1.9%, 1.7% and 1.5% respectively.

**Conclusions:**

A range of challenges towards good mental health are portrayed, including diagnostic limitations, poor awareness on MD, and socioeconomic inequality. It is recommended that these challenges are tackled, and routine screening and early intervention services are developed to improve early identification and prompt treatment. Achieving these goals may positively impact individuals and societies at large, both now and in the future.

